# Data Collection, Modeling, and Classification for Gunshot and Gunshot-like Audio Events: A Case Study

**DOI:** 10.3390/s21217320

**Published:** 2021-11-03

**Authors:** Rajesh Baliram Singh, Hanqi Zhuang, Jeet Kiran Pawani

**Affiliations:** 1 EECS Department, Florida Atlantic University, Boca Raton, FL 33431, USA; zhuang@fau.edu; 2 ECE Department, Georgia Tech, Atlanta, GA 30332, USA; jpawani3@gatech.edu

**Keywords:** gunshot, plastic bag pop, binary classification, convolution neural network, Mel-frequency cepstral coefficients, Mel-spectrogram

## Abstract

Distinguishing between a dangerous audio event like a gun firing and other non-life-threatening events, such as a plastic bag bursting, can mean the difference between life and death and, therefore, the necessary and unnecessary deployment of public safety personnel. Sounds generated by plastic bag explosions are often confused with real gunshot sounds, by either humans or computer algorithms. As a case study, the research reported in this paper offers insight into sounds of plastic bag explosions and gunshots. An experimental study in this research reveals that a deep learning-based classification model trained with a popular urban sound dataset containing gunshot sounds cannot distinguish plastic bag pop sounds from gunshot sounds. This study further shows that the same deep learning model, if trained with a dataset containing plastic pop sounds, can effectively detect the non-life-threatening sounds. For this purpose, first, a collection of plastic bag-popping sounds was recorded in different environments with varying parameters, such as plastic bag size and distance from the recording microphones. The audio clips’ duration ranged from 400 ms to 600 ms. This collection of data was then used, together with a gunshot sound dataset, to train a classification model based on a convolutional neural network (CNN) to differentiate life-threatening gunshot events from non-life-threatening plastic bag explosion events. A comparison between two feature extraction methods, the Mel-frequency cepstral coefficients (MFCC) and Mel-spectrograms, was also done. Experimental studies conducted in this research show that once the plastic bag pop sounds are injected into model training, the CNN classification model performs well in distinguishing actual gunshot sounds from plastic bag sounds.

## 1. Introduction

As cities become more densely populated, the volume of criminal activities follows suit [1,2]. It has become a sine qua non measure that false detections generated by existing gunshot detection systems are reduced to a minimum. The needless deployment of public safety personnel can result in a situation in which their presence is needed in a life-threatening scenario when they are otherwise occupied responding to a false alarm, like a plastic bag explosion.

The ability of a model to correctly discern sounds, even in the subtlest of scenarios, will differentiate a well-trained model from one that is not very efficient. In addition to a high true positive rate (TPR), a good measure for judging a detection system is its false positive rate (FPR). A well-designed system will keep this value close to zero in addition to pushing the value of TPR to one.

Over the past few years, there has been a degree of hesitation over the implementation of some of the well-known available acoustic gunshot detector systems (AGDS). Some of the factors affecting the implementation of these detection systems include the price point of the service and the reliability of the systems. This paper will focus more on addressing the reliability aspect of the AGDS as it relates to the FPR. The motivation of this work stems from the fact that datasets containing sounds closely related to gunshot sounds are not readily available for research purposes. Although a great amount of work has been presented in the detection of gunshot sounds [3,4,5,6,7,8,9,10,11,12,13,14,15,16], comparative analyses of similar audio events are not as detailed, though in the literature, researchers carried out comparative analyses of similar sounding events to gunshot audio events with abrupt changes in energy, such as door slams, claps, and firecrackers [10,11,12,13,14,15,16].

For the research carried out in [11], a 97.62% TPR was achieved for gunshot recordings, door slams, ticks, and human voice for an eightfold cross-validation experiment. The work in [16] provided the comparison of impulsive sounds which includes the explosion of a balloon, clap and the sound of plosive consonants such a [p], [t], and [k]. The authors of [10,11,12,13,14,15,16] all discuss the detection of gunshot sounds in comparison to other sounds; however, they did not provide a database of the non-gunshot sounds readily available for further research.

In this paper, our research indicate that fake gunshot sounds can easily confuse a gunshot sound detection model. To approach this problem, we detail our effort to create a dataset of plastic bag pop sounds, a type of sound that can easily be misclassified as gunshot sounds. To underscore the confusion of gunshot and gunshot-like sounds, there was a case where students who survived a school shooting, thought that the gunshots fired in the school were actually plastic bag pops [17]. This is a case where actual gunshots were mistaken for plastic bag pops. We also develop a simple binary classification model based on a convolutional neural network (CNN), which served as the baseline for the purpose of assessing the performance of a gunshot detection model. The remainder of the paper is organized in the following manner. Section 2 gives the details on how samples of plastic pop sounds are measured. Section 3 outlines the experimental setup adopted in this research. The results and discussion are presented in Section 4. The paper ends with concluding remarks in Section 5.

## 2. Data Collection

As a parallel to the work discussed in [18], we created a new dataset comprised of audio recordings of plastic bag explosions collected over a variety of environments and conditions.

It has been observed that, as humans, we use additional sensory inputs and past experiences to identify sounds [19]. Computers, on the other hand, are trained to decipher information that is often irrelevant or imperceptible to human ears. On this thread, every fragment of audio information is valuable for a sound recognition system to do its job effectively. In gunshot detection, a database of a particular type of sound that can be confused with gunshot sounds but is rich in diversity can lead to a more effective gunshot detection system [20]. This was the motivation in creating a database of plastic bag explosion sounds.

We designed multiple experiments to collect plastic bag pop sounds, taking into consideration various factors such as the sound environment, plastic bag materials, recording devices, and distances between sound sources and recording devices. In addition, to create a user-friendly sound dataset, we carefully selected the software tools to process sounds and designed a file naming convention.

### 2.1. Environment

Environmental conditions play a crucial role in recording sounds for evaluating algorithms and models designed for sound detection and classification. Similar to how bats swoop around objects as they transmit high-pitched sound waves that will bounce back to them at different time intervals [21], we use different environments to give the machine learning (ML) algorithm a better perception sense of the differentiation of the closely related sounds. We can look at the former scenario as changes in the stimuli to determine the environment, while looking at the latter scenario as changes in the environment to determine the stimuli. The higher the diversity of the same sound, the higher the likelihood that the ML algorithm will correctly detect that specific sound.

**Figure 1:**
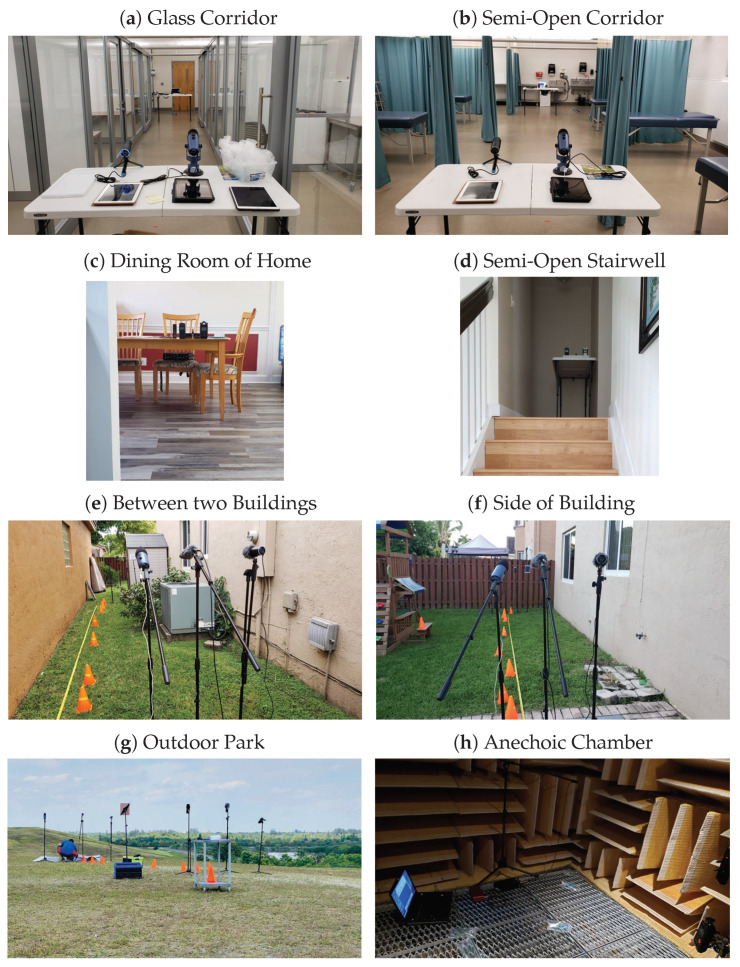
Images of the various environments used.

Gunshot-like sounds were recorded in the locations where there was a likelihood of guns being fired, the authors chose indoor and outdoor locations. Mixed in with common locations, an ideal or distortion-free location was also included. To this end, the authors chose eight environments: (1) inside a building, along a corridor with glass walls; (2) inside a building, along a corridor with curtains and beds; (3) inside the dining room of a home; (4) along a half-opened stairwell in a home; (5) outdoors between two buildings; (6) outdoors on the side of a building; (7) outdoors, in an open field; and (8) in an anechoic chamber. Figure 1 below shows pictures of the actual setups and environments in which the data was collected.

Although it will take a very long time to capture data on a multitude of surroundings, the environments mentioned above are representative to expose the ML algorithm to a wide range of features.

**Figure 2:**
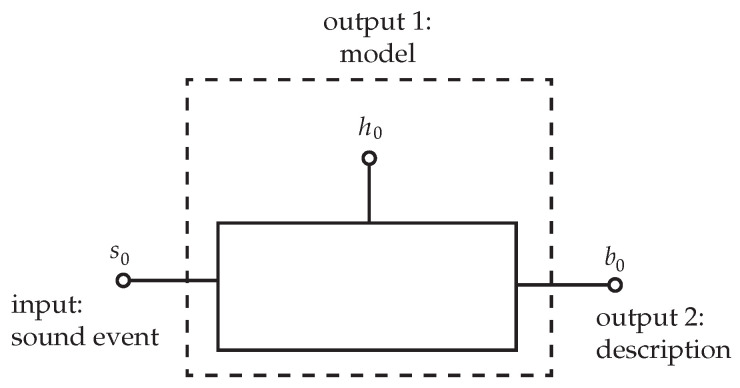
Schematic representing an auditory experiment.

A simple schematic representative of the basic operation taking place during an auditory event [22] is shown in Figure 2 below. Although this schematic is used to describe spatial attributes, it does give us a good visual for gaining a better understanding of the ML process as it relates to our proposed sound database.

As we feed in a variety of input sounds ($s_{0}$), the model ($h_{0}$) will continually learn and eventually be able to correctly differentiate the nuances of the input to give a correct output ($b_{0}$), just as a child to discover how to discern sounds by learning from their surroundings, listening, and then seeing or experiencing. We humans can distinguish between, for example, a plastic bag rustling in the wind and a metal can blowing around the ground.

As mentioned earlier, different surroundings allow for a wide range of features that can be extracted from the audio files. Section 2.5 below provides a good visual representation of this idea.

### 2.2. Plastic Bag Material

The data collection process started with experimentation with various types of bags. After this experimentation with different bags—such as those from the supermarket and department store, as well as trash can liners—it was found that the most suitable bags were those used as trash can liners. The other bags would burst along their seams, resulting in a poor audio representation of a plastic bag explosion.

Trash can liners have an added benefit: different sizes can be easily procured. Various trash can liner sizes were used: 1.89, 3.03, 9.08, and 15.14 liters.

### 2.3. Distances

**Figure 3:**
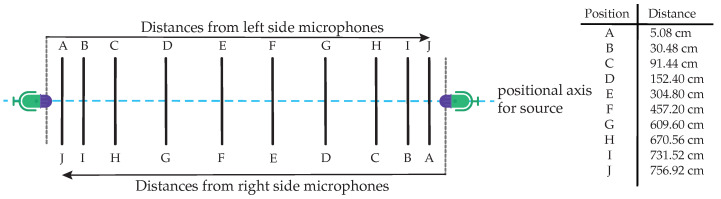
Various distances from left and right microphones.

Various distances from the sound sources to the microphones were also used as a variable in this data collection process. Figure 3 below shows the distances used to capture the audio samples. The plastic bag explosions would begin on the right side, where there were the Tascam DR-05X, Zoom H4nPro, and Brüel & Kjær (type 2671) microphones. On the left side, there were the following recording devices: Blue Yeti USB, JLab TALK GO, Samsung S9, and an iPad mini.

Carrying out this procedure saved some time, as only ten bags had to be filled and then popped. Moving from right to left and using just ten bags, twenty distance-related audio clips were captured.

### 2.4. Recording Devices

Most of the audio clips were captured using the six aforementioned recording devices: Tascam DR-05X, Zoom H4nPro, Brüel & Kjær, Blue Yeti USB, JLab TALK GO, and a Samsung S9. A seventh recording device, an iPad mini, was added and used only for capturing the outdoor audio. These different recording devices allowed for the capture of plastic bag pop sounds with various audio responses and audio levels.

### 2.5. Spectrogram Plots

**Figure 4:**
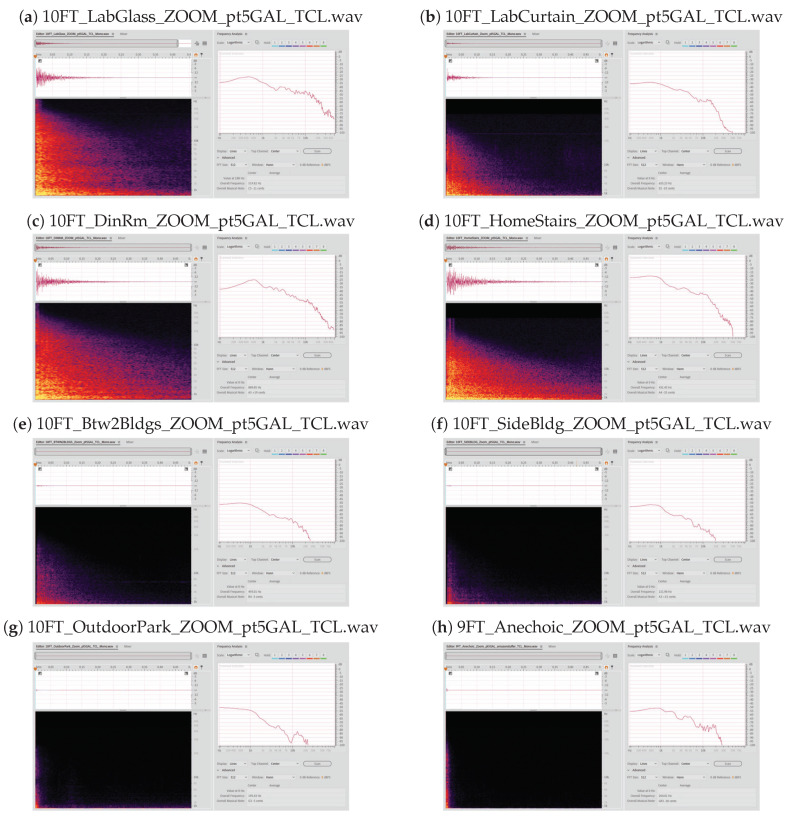
Spectrogram plots for the different environment of the Zoom microphone.

As we bring together all the variables discussed above together, we observe, for a single group of variables, the spectrogram plots shown in Figure 4 below. The spectrogram plots are derived from varying the environment of the same size plastic bag (the 1.89 liter bag, in this case), with each audio file taken at 304.80 cm (10 ft) from the Zoom microphone. Each plot was captured in Adobe Audition.

Figure 4 above was taken (**a**) indoors along a glass corridor, (**b**) indoors along a corridor with curtains and hospital beds, (**c**) indoors in a dining room of a home, (**d**) inside a home with a half-opened stairwell, (**e**) outdoors between two buildings, (**f**) outdoors on the side of a building, (**g**) outdoors in a park, and (**h**) in an anechoic chamber.

Each plot in Figure 4 above shows the corresponding frequency response to the right of the spectrogram plot. Note that the corresponding frequency response was calculated using an FFT size of 512 with a Hann window. For the sake of keeping the file names small, the SI units were converted to metric (details of the file naming convention is found in Section 2.7 below).

**Figure 5:**
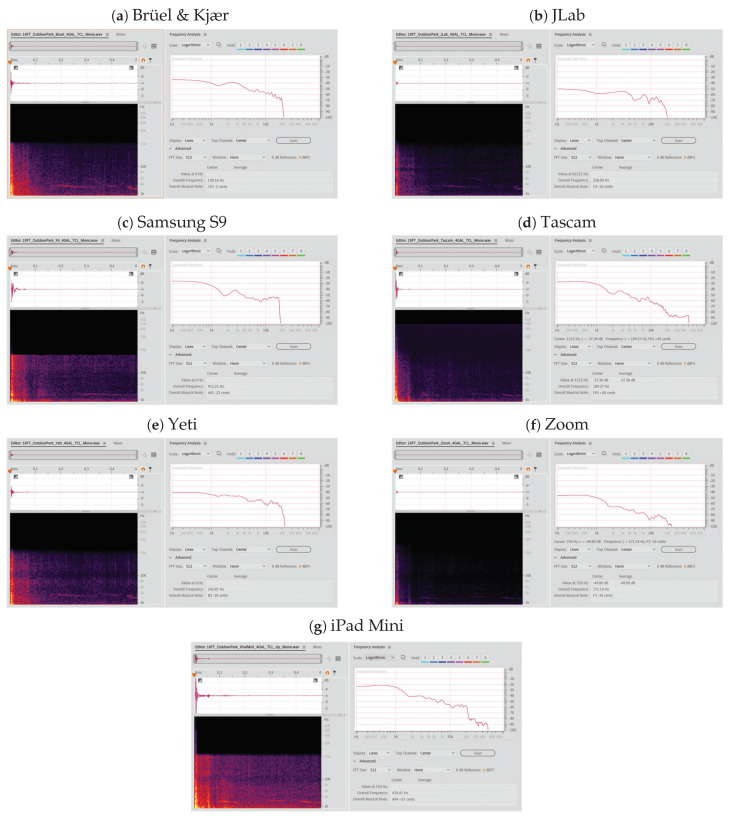
Spectrogram plots with the frequency response for the 15.14 liters bag at 304.80 cm from the various microphones in the outdoor park.

Figure 5, below, shows the spectrogram plots in the same environment (outdoor park), with the data taken at 304.80 cm from the various microphones of the 15.14 liters plastic bag.

### 2.6. Software

Adobe Audition CC 14.4.0.38 was the main software utilized during the editing and saving of the final cut audio clips. The editing consisted of cutting the audio clip down to approximately 500 ms with a 24-bit depth and a 192 kHz sample rate. Some of the audio clips captured were recorded in stereo, while others were recorded in mono (Brüel & Kjær). To “standardize” the audio clips being fed into detection and classification algorithms, all the audio clips were passed through the Sound eXchange (SoX) software [23] to extract just Channel 1 from the audio.

A secondary software, Sound Forge 14 Pro Suite with the Steinberg Spectral Layers Pro 7 was also used during the initial stages of the database development. This software gave a slightly different interface such that the spectral components can be viewed in a 3D environment.

### 2.7. File Naming Convention

In line with the Urban Sound dataset 8 k [24], the files were named depending on their distance, location, and size. For example, an audio clip taken for the 9.08 liter [2.4 US gallon] bag (*204*) 91.44 cm [3 ft] away (*300*) from the S9 recording device (*06*) in the curtain environment (*02*) was originally named *3FT LabCurtain S9 2pt4GAL TCL.wav*. After application of a value naming convention (numbers in brackets), that specific file was renamed to *300020620400.wav*. The last two digits signify whether the audio was unclipped (*00*), lightly clipped (*01*), or heavily clipped (*02*). This filename was then used in the metadata .csv file that was part of the Python code to get the associated labeled data class. Note that all the plastic bag explosion audio files used in the analysis were unclipped to maintain a spectrally pure analysis of the new dataset. Table 1 below lists the quantity of the un-clipped and lightly clipped audio files currently in the database.

## 3. Experimental Setup

### 3.1. Dataset Organization

The audio clips collected using the measures described in Section 2 above were then used to develop a classification model based on a CNN network. The 374 gunshot samples initially used to train the model were obtained from the Urban Sound dataset [24]. All other sounds were deleted from the original dataset and only the gunshot samples were kept. Meanwhile, an equivalent number of plastic bag explosion sounds from the new proposed database was added to each fold, ensuring a balanced dataset.

To ensure that there is no leakage of data from the training dataset to the testing dataset, out of the 748 dataset samples (374 gunshot and 374 plastic bag explosion), 13% (or 100 samples) was reserved for testing; this was not used in the training phase, but instead was used only once to verify the final model. The remaining 648 were randomly divided into two subsets: 70% for training and 30% for validation. Details on how well the model performed can be found in Section 4 below.

### 3.2. Classification Model

**Figure 6:**
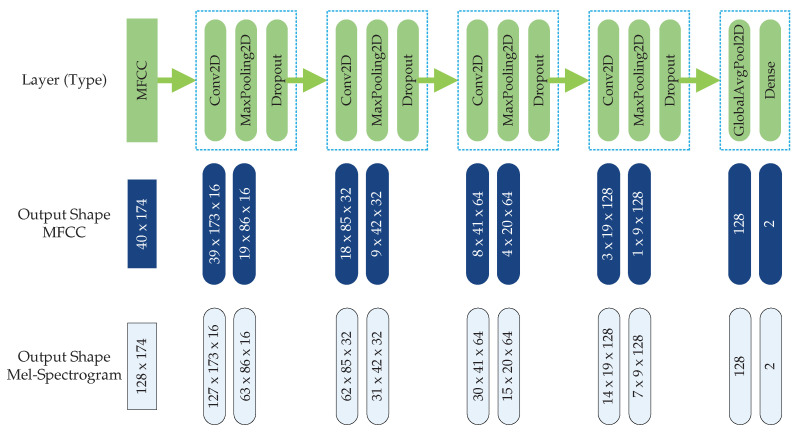
CNN architecture used in classification of gunshot sounds for both MFCC and Mel-spectrogram features.

To set a baseline for how a classification model will behave when it is trained and tested with the newly created sound dataset, a simple four-layer CNN model using softmax as the classifier was employed. Figure 6 below shows the CNN architecture, as well as the output dimensions of the sound signals as they pass through each layer. In both cases, i.e., the Mel-frequency cepstral coefficients (MFCC) and the Mel-spectrogram, the number of trainable parameters totaled 43,570.

## 4. Results and Discussion

To check on the extent of which a sound classification model could be confused by fake gunshots, we trained the model without letting it be exposed with plastic bag pop sounds. To this end, we first used the CNN model shown in Figure 6 above with the MFCC features on the original Urban Sound 8k dataset with ten classes (gun_shot, dog_bark, children_playing, car_horn, air_conditioner, street_music, siren, engine_idling, jackhammer, and drilling). After training, the model was used to test its ability to reject plastic bag pop sounds as true gunshot sounds. It was found that 75% of the plastic bag pop sounds were misclassified as gunshot sounds. The high percentage of misclassification indicates that it is very difficult for a classification model to discern gunshot-like sounds such as plastic bag pop sounds from real gunshot sounds. This warrants the process of developing a dataset containing sounds that are similar to real gunshot sounds.

**Figure 7:**
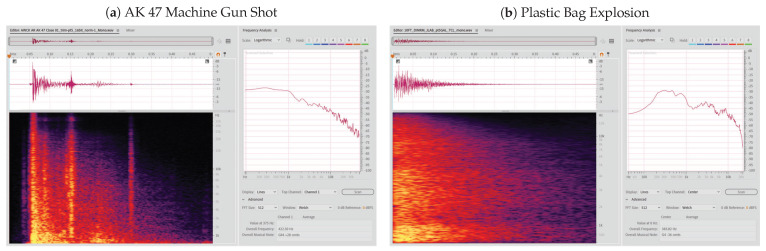
Comparison ofspectrogram plots between (**a**) an AK47 gunshot and (**b**) a plastic bag explosion.

Figure 7 below compares the Mel-spectrogram between an AK 47 gunshot and a plastic bag explosion as displayed on Adobe Audition CC 14.4.0.38. The plastic bag explosion with its spectrogram shown in Figure 7b was recorded on the JLab microphone in the dining room of a home. The spectrogram plots had an FFT size of 512 using the Welch window. Note that the peaks found in Figure 7a is from the clip action of the machine gun.

**Figure 8:**
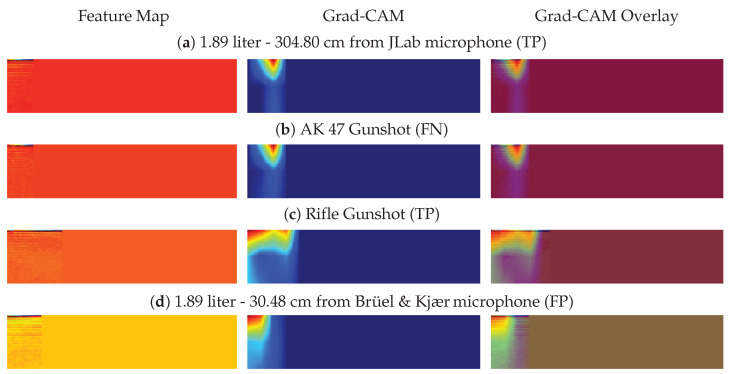
Comparison of the Grad-CAM visualization for the MFCC feature extraction between plastic bag explosions and gunshots.

To show that plastic bag explosions can present problems even to the CNN model, we applied the Gradient-weighted Class Activation Mapping (Grad-CAM) visualization technique to generate heat maps of the gunshot and gunshot-like features [25], as illustrated in Figure 8 below.

As can be observed from Figure 8, the CNN can mistake a gunshot sound for a plastic bag explosion by the manner in which the network looks at the feature maps. Figure 8a shows where the CNN localizes its class-discriminative regions for the plastic bag explosion case, and in turn localizes around the same regions for the AK 47 gunshot case as shown in Figure 8b. This operation results in the CNN generating a FN as it classifies the AK 47 gunshot as a plastic bag explosion during the MFCC feature extraction case. A similar reasoning can be applied for the case where the plastic bag explosion sound (Figure 8d) can be confused for a gunshot sound (Figure 8c).

**Figure 9:**
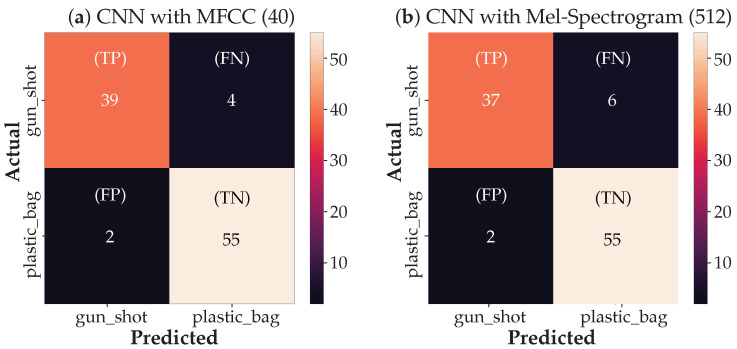
Normalized Confusion Matrix with: (**a**) 40 MFCC Coefficients and (**b**) Mel-spectrogram with FFT window = 512.

Next, we present the experimental results using gunshot sounds and plastic bag pop sounds together to train and test the CNN classification model. The confusion matrices (CMs) for both the MFCC and Mel-spectrogram feature extraction analysis are shown in Figure 9 below. The CMs were generated from a sample of 100 audio clips (never seen before by the model) randomly held back from the database as discussed in Section 3.1 above.

The CMs show that the true positives (TP), i.e., events correctly predicted to be actual positives, for the gunshot sound are 39 and 37 from the CNN using the MFCC and Mel-spectrogram features, respectively. The true negative (TN) events correctly predicted as actual negatives are 55 for both the MFCC and the Mel-spectrogram cases. A false positive (FP) or type-I error occurs when the actual value was negative but the model predicted a positive value, 2 in both cases. The false negative (FN), a type-II error, occurs when the actual value was positive but the model predicted a negative value, i.e., 4 and 6 for the MFCC and Mel-spectrogram cases, respectively.

Table 2 below shows different metrics comparing the MFCC and Mel-spectrogram cases. While the sensitivity is a measure of the proportion of the actual positive cases that were correctly predicted, specificity is the measure used to determine the proportion of correctly predicted negative cases. Specificity can be considered complementary to the false positive rate (FPR). The precision and F1 score (or F-measure) can also be seen in Table 2 below, where precision is the ratio of relevant events to the total events, and the F1-score is the harmonic mean of precision and sensitivity.

**Figure 10:**
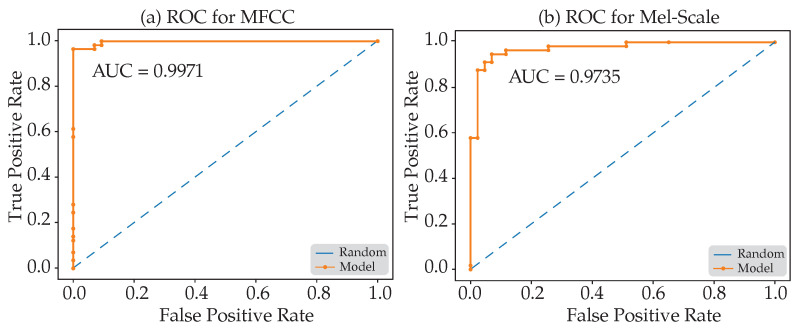
ROC curves for (**a**) MFCC and (**b**) Mel-spectrogram feature extraction.

From the overall metrics, the CNN classifier using the MFCC features as inputs appears to be performing better than the model using the Mel-spectrogram features. By inspecting the receiver operating characteristic curve (ROC) shown in Figure 10 below, we notice that the classifier with the MFCC feature extraction attains 0.9971 for the area under the curve (AUC), which is better than that of the Mel-spectrogram case.

## 5. Conclusions

The work in this paper presented a new sound dataset consisting of a series of plastic bag explosion sounds. These explosion sounds closely match gunshot sounds. The main aim of this data collection was to improve the performance of a gunshot detection algorithm, in particular, to reduce its false positive rate, i.e., to reduce the chances of treating innocuous audio trigger events as perilous audio events involving firearms. We also developed a simple classification algorithm, as a baseline, to illustrate the relevance of this data collection effort. It is understood that it may be a daunting task to account for all sounds that are similar to a gunshot sound. Arranging binary classification of various types of gunshot-like sounds vs. real gunshot sounds, one type at a time, in a hierarchical classification scheme, it is foreseen that a classification model can be developed to achieve satisfactory performance in separating all types of fake gunshot sounds from true gunshot sounds. This dataset, along with the classification model trained for gunshot and gunshot-like sounds, in turn, is a step leading to much fewer false positives and in improving overall public safety by deploying critical personnel only when necessary.

## Figures and Tables

**Table 1:** Number of audio files according to their clipping state.

Clipping	# Audio Files
No Clip	786
Light Clip	160

**Table 2:** Comparison of scoring data between the MFCC and Mel-spectrogram feature extraction.

	MFCC	Mel-Spectrogram
**Sensitivity**	0.9070	0.8605
**Specificity**	0.9649	0.9649
**Precision**	0.9512	0.9487
**F1 Score**	0.9285	0.9024
**AUC**	0.9971	0.9751

## Data Availability

The full dataset used can be found at https://github.com/rbsingh13/Plastic-Bag-Pop-sounds, accessed on 31 October 2021. Please cite this article if you use this dataset for your research.

## Abbreviations

The following abbreviations are used in this manuscript:

AGDS	Acoustic Gunshot Detector Systems
AUC	Area Under the Curve
CM	Confusion Matrix
CNN	Convolution Neural Network
FN	False Negative
FP	False Positive
FPR	False Positive Rate
Grad-CAM	Gradient-weighted Class Activation Mapping
MFCC	Mel-Frequency Cepstral Coefficients
ML	Machine Learning
ROC	Receiver Operating Characteristic
TN	True Negative
TP	True Positive
